# Prospects for Expansion of Universal Newborn Screening in Bulgaria: A Survey among Medical Professionals

**DOI:** 10.3390/ijns9040057

**Published:** 2023-10-11

**Authors:** Georgi Iskrov, Vyara Angelova, Boyan Bochev, Vaska Valchinova, Teodora Gencheva, Desislava Dzhuleva, Julian Dichev, Tanya Nedkova, Mariya Palkova, Anelia Tyutyukova, Maria Hristova, Eleonora Hristova-Atanasova, Rumen Stefanov

**Affiliations:** 1 Department of Social Medicine and Public Health, Faculty of Public Health, Medical University of Plovdiv, 15A Vasil Aprilov Blvd., 4002 Plovdiv, Bulgaria; georgi.iskrov@mu-plovdiv.bg (G.I.); rumen.stefanov@mu-plovdiv.bg (R.S.); 2 Institute for Rare Diseases, 22 Maestro G. Atanasov St., 4017 Plovdiv, Bulgaria; 3 Faculty of Medicine, Medical University of Plovdiv, 15A Vasil Aprilov Blvd., 4002 Plovdiv, Bulgaria; vyara.plovdiv@gmail.com (V.A.); bojan_bo@abv.bg (B.B.); vasi.val@abv.bg (V.V.); tedgencheva@gmail.com (T.G.); desislava.dzhuleva@gmail.com (D.D.); julian.dichev@abv.bg (J.D.); tanyanedkova26@gmail.com (T.N.); palkovamariya2001@gmail.com (M.P.); anelia2001t@gmail.com (A.T.); mariaghristova11@gmail.com (M.H.)

**Keywords:** newborn screening, neonatal screening, Wilson–Jungner principles, rare diseases, screening panel, Bulgaria

## Abstract

Determining the scope of a newborn screening program is a challenging health policy issue. Our study aimed to explore the attitudes of specialists in pediatrics, neonatology, medical genetics, and biochemistry regarding the prospects for expanding the panel of diseases for universal newborn screening in Bulgaria. We conducted an online survey in March–May 2022. The questionnaire listed 35 disorders that could potentially be included in the Bulgarian panel for universal newborn screening. If endorsing a specific condition, participants had to justify their position by judging its performance against the ten principles of Wilson and Jungner. We found a high degree of knowledge about the current universal newborn screening program in Bulgaria. An overwhelming majority (97.4%) supported the expansion of the panel to include more conditions. Four disorders obtained more than 50% approval for inclusion: cystic fibrosis (87.0%), thalassemia (72.7%), spinal muscular atrophy (65.6%), and classical galactosemia (59.1%). The perception of the condition as an important health problem was the most significant factor in this support. The costs of diagnosis and treatment appeared to be the main source of concern. We recommend country-specific economic evaluations and research on the views of other stakeholders, including the government, payers, and patient organizations, to better understand and manage the complex nature of newborn screening policymaking.

## 1. Introduction

Newborn screening (NBS) is a means of secondary prevention that entails testing newborn babies shortly after birth to find specific disorders that are not apparent but might result in major health problems if left untreated. The goal of NBS is to detect these conditions early so that effective medical intervention can be provided to prevent or minimize the onset of health consequences [1,2]. Determining the scope of NBS is a challenging health policy issue [3]. In 1968, the World Health Organization (WHO) released a milestone paper on the “Principles and Practice of Screening for Disease” by Wilson and Jungner [4]. This report outlined the settings under which screening could be justified as a public health strategy [5,6,7].

Following the success of the first universal NBS for phenylketonuria in the 1960s, tests for other conditions were gradually added to NBS programs [8,9,10]. This expansion varied across different countries, depending on their healthcare systems and policy priorities, as well as the availability of human, physical, and financial resources [1,3,7]. The principles of Wilson and Jungner are often invoked to inform these decisions. However, insights from local stakeholders, consideration of effectiveness and cost effectiveness, and adaptation to the country’s context are also sought [7]. In the European Union (EU) Member States, the number of specific conditions included in the universal NBS panel currently ranges from two to thirty-six diseases, with a clear trend for an increase over the last decade [9].

Bulgaria is among the EU countries with the most limited NBS panel [1,2]. The Minister of Health’s Ordinance No. 26 of June 14, 2007, on providing obstetric care to women without health insurance and on conducting examinations and tests outside the scope of mandatory health insurance for children and pregnant women mandates universal NBS. The Ministry of Health is funding—outside the scope of compulsory health insurance—the universal NBS of all newborn children for phenylketonuria, congenital adrenal hyperplasia, and congenital hypothyroidism [11]. There are two national reference laboratories: the National Genetic Laboratory at the University Obstetrics and Gynecology Hospital “Maichin Dom” (NBS for phenylketonuria) and the Screening and Functional Endocrine Diagnostics Laboratory at the University Pediatric Hospital “Professor Ivan Mitev” (NBS for congenital hypothyroidism and congenital adrenal hyperplasia) [2,9]. All subsequent diagnostic activities are reimbursed by public funds [2,11].

Over the past fifteen years, there have consistently been discussions—both official and unofficial—about broadening the list of disorders for universal NBS in Bulgaria. The aim of this study is to explore the attitudes of specialists in pediatrics, neonatology, medical genetics, and biochemistry regarding the prospects for expanding the panel of diseases for universal NBS in Bulgaria.

## 2. Materials and Methods

Methods are presented in compliance with the Checklist for Reporting Results of Internet E-Surveys, CHERRIES (Supplementary File S3) [12].

### 2.1. Survey Sample

Survey participants included specialists in pediatrics, neonatology, medical genetics, and biochemistry from Bulgaria. The selection of this target group was based on the state-of-the-art of NBS in Bulgaria. These healthcare professionals are involved in NBS activities and in the subsequent diagnostic confirmation, treatment, and follow-up of detected cases. While the expansion of the current NBS program is a matter of political decisions, the experience and expertise of this community are likely to inform and advise Bulgarian policymakers.

A purposive sample was drawn from Bulgarian healthcare professionals with publicly available email addresses. The criteria for inclusion were the specialty of pediatrics, neonatology, medical genetics, and/or biochemistry, and the affiliation with a healthcare provider in this field. We specifically focused on the university hospitals (as the largest tertiary healthcare centers in the country), as well as the Bulgarian Pediatric Association and the Bulgarian Society of Human Genetics and Genomics.

We identified a total of 338 individuals and contacted them via email to participate in the online survey with an invitation letter describing the study, including the purpose and investigators, the number of questions, the estimated time to complete the survey, and the data storage modality used. No incentives for participation were provided.

Ethical approval was not required for this study in accordance with the national and institutional guidelines. The survey was sociological from a methodological point of view, with no clinical research.

### 2.2. Survey Items

The questionnaire consisted of 46 questions grouped into four sections: socio-demographic and career profile (6 items); knowledge and attitudes towards the current NBS program in Bulgaria (5 items); attitudes towards the expansion of universal NBS in Bulgaria with additional disorders to screen (35 items).

The third section of the questionnaire listed 35 disorders or groups of disorders that could be potentially included in the Bulgarian panel for universal NBS. This set was based on the study of Loeber et al. in 2021, which listed a total of 38 conditions that are currently included in at least one of the national panels for universal NBS in the EU [9]. Three of them—phenylketonuria, congenital adrenal hyperplasia, and congenital hypothyroidism—are already included in the Bulgarian panel, so participants were asked about the remaining conditions.

Adaptive questioning was used in this section of the survey. If a respondent agreed that a certain disorder or group of disorders should be added to the NBS, they had to explain why by comparing how well the condition worked with the ten principles of Wilson and Jungner [4]. Both the list of disorders and the list of Wilson–Jungner principles were randomized in order to minimize the order bias in the survey.

The questionnaire was piloted among a small group of medical professionals to improve the consistency and clarity. One modification was made at this point. NBS for hemoglobinopathies usually targets sickle cell disease and thalassemia. Experts suggested narrowing the scope only to thalassemia for practical reasons. The final set of 35 prospective disorders to screen is listed in Supplementary File S1.

### 2.3. Electronic Survey Properties

We started the online survey on 24 March 2022, and then sent weekly reminders. The survey was active until 31 May 2022. Electronic consent was obtained after reading the study’s description and before launching the questionnaire.

We used an open-mode electronic survey. However, the link to complete the questionnaire was not publicly posted or available anywhere. There was neither a public announcement nor an advertisement. We only disseminated the information about the survey via emails to the predefined sample of 338 individuals. IP checks were conducted to reduce the likelihood of duplicate entries coming from the same respondent. Cookies and other techniques for identifying multiple entries were not applied.

Respondents were able to review and change their answers at any time. All survey items were mandatory to complete, with an optional free text field after each question for providing additional input. A completeness check was automatically conducted before submitting each section of the questionnaire.

No personal information was collected or saved. All the gathered data were stored on a protected server with controlled access.

### 2.4. Data Analysis

Partially completed questionnaires were discarded. Descriptive statistics were applied. We used the chi-square test twice: to look at the relationship between the type of measure to improve NBS and how important it was thought to be, and to look at the relationship between the disorder to screen for and how well it did on the Wilson–Jungner criteria. Statistical significance was considered if the *p*-value was less than 0.05. The data were analyzed using Microsoft Excel 365.

## 3. Results

### 3.1. Socio-Demographic and Career Profile of the Respondents

A total of 183 invitees started the questionnaire (a participation rate of 54.1%). Twenty-nine of them did not finish the survey, and their responses were not included in the subsequent analysis. Thus, 154 participants fully completed the questionnaire (a completion rate of 84.2%).

The socio-demographic and career profile of the respondents is presented in Table 1. The sample demonstrated a high level of qualification and experience. The mean professional experience was 21.5 ± 13.2 years. Half of the participants (*n* = 83, 53.9%) had a Ph.D. degree, and 19 (12.3%) had a D.Sc. degree; 93 respondents (60.4%) indicated having more than one medical specialty.

### 3.2. Knowledge and Attitudes towards the Current NBS Program in Bulgaria

The study sample reported a high degree of knowledge about the current universal NBS program in Bulgaria, even though 85 participants (55.2%) reported no direct involvement in it (Table 2). One-third of the respondents (*n* = 48, 31.2%) indicated participation in the treatment and follow-up of diagnosed cases, while 18.2% (*n* = 28) were engaged in the confirmation of diagnosis.

**Figure 1:**
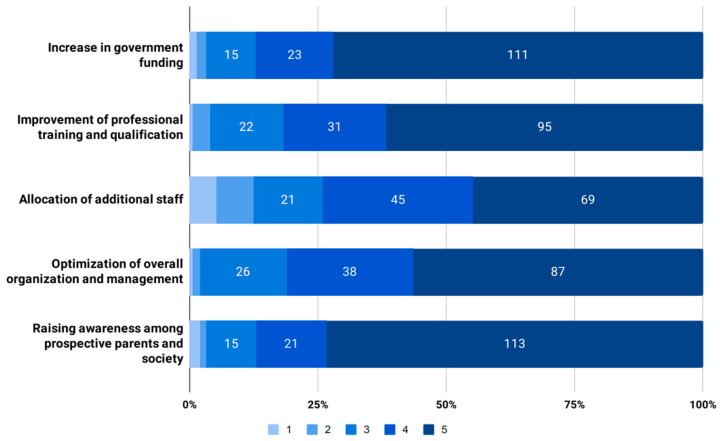
Measures to improve the outcomes of the current universal NBS program in Bulgaria assessed on a 1–5 scale (1 being the least important and 5 being the most important).

The outcomes of the current universal NBS program were positively appraised. Thirty-nine (25.3%) gave the highest grade, with just one respondent giving them the lowest mark (Table 2). When the respondents were asked to assess potential measures to improve the results of the current program, raising awareness among prospective parents and society as a whole and increasing government funding were considered the most important (*p* = 0.00). Allocating additional staff was deemed the least important (Figure 1). An overwhelming majority (*n* = 150, 97.4%) supported the expansion of the panel to include more conditions for universal NBS in Bulgaria.

### 3.3. Attitudes towards the Expansion of Universal NBS in Bulgaria with Additional Disorders to Screen

**Figure 2:**
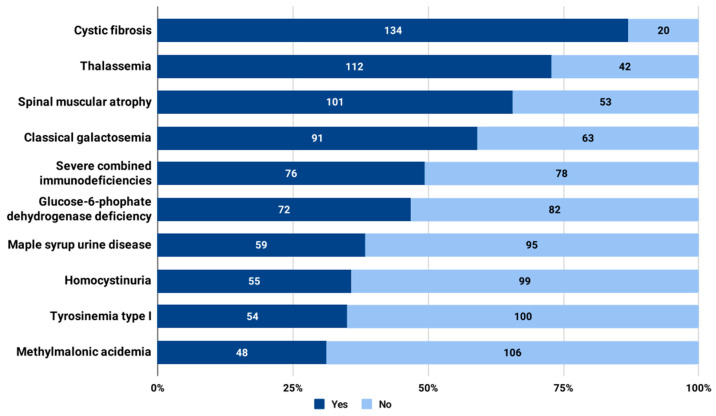
Approval of inclusion in the panel for universal NBS in Bulgaria for the top 10 disorders.

Four diseases obtained more than 50% approval to be added to the panel for universal NBS in Bulgaria: cystic fibrosis (*n* = 134, 87.0%), thalassemia (*n* = 112, 72.7%), spinal muscular atrophy (*n* = 101, 65.6%), and classical galactosemia (*n* = 91, 59.1%) (Figure 2). Severe combined immunodeficiencies (*n* = 76, 49.4%) and glucose-6-phophate dehydrogenase deficiency (*n* = 72, 46.8%) came next. Eight conditions obtained support of between 20% and 30%, and seventeen between 10% and 20%. Methionine adenosyl transferase I/III deficiency received the lowest support among the respondents, at 11.7% (*n* = 18). The full set of approval rates for the 35 prospective disorders to screen is listed in Supplementary File S2.

Using the principles of Wilson and Jungner, we found a consistent, similar pattern behind the rationale for the approval of the top three disorders, *p* = 0.0852 (Table 3). The perception of the condition as an important health problem was the most significant factor for support: cystic fibrosis (*n* = 117, 87.3%), thalassemia (*n* = 94, 83.9%), and spinal muscular atrophy (*n* = 91, 90.1%). On the other hand, the costs of diagnosis and treatment appeared to be the main source of concern.

## 4. Discussion

### 4.1. Prospective Conditions to Be Added to the Universal NBS Panel

NBS for cystic fibrosis is currently a well-established public health approach with international standards [13]. Over the last decade, there has been a clear uptake in adopting and launching this initiative in an increasing number of jurisdictions. In 2016, a survey found that there were 17 national and 4 regional NBS programs for cystic fibrosis in Europe [13]. In 2022, there were already 22 national and 34 regional programs of this type [14]. This landscape obviously makes cystic fibrosis the most prospective condition to be added to the Bulgarian NBS panel. Bulgarian cystic fibrosis patients have long remained a vulnerable population, with risk factors for worse health outcomes [15]. There have been calls by local experts to introduce NBS for this rare condition in the country [16]. Nevertheless, as other authors also suggest, the lack of financial resources and political inertia seem to be the main challenges [14].

Thalassemia came in second in terms of approval for inclusion in the Bulgarian panel for universal NBS. Thalassemias are a group of inherited hemoglobin disorders [17]. Bulgaria is in a geographical region where this group of disorders is relatively common [18]. It is estimated that the prevalence of beta-thalassemia carriers in the country’s population is about 2.5% [19]. Despite this, those conditions are not currently part of any mandatory screening activities. In addition to NBS, thalassemia could be subject to antenatal screening and testing of pregnant women as well [20]. These public health interventions provide couples with information about their reproductive risk and allow them to make informed reproductive choices [21]. Nevertheless, the current NBS programs for hemoglobin disorders are seen as valid and easy to perform, enabling early diagnosis and comprehensive care [17,22,23].

Similar conclusions could be drawn about the third-placed condition from our study, spinal muscular atrophy. Compared to the previous two disorders, NBS for spinal muscular atrophy is a relatively new public health activity due to the recently approved disease-modifying therapies [24]. Otherwise, this rare disease has been considered incurable in the past [25]. Reports of early experiences with NBS for spinal muscular atrophy describe major clinical utility to support parental decision-making as well as to facilitate access to specialist care [26]. Experts agree that interest in this NBS program will steadily increase in the years to come, especially in jurisdictions where novel medicinal therapies for this condition are available [27,28]. From that point of view, both nusinersen and risdiplam are reimbursed in Bulgaria [29], so this prerequisite is met if NBS for spinal muscular atrophy is to be implemented in the country.

Finally, classical galactosemia was ranked fourth in terms of approval for inclusion in the panel for universal NBS in Bulgaria. This is an interesting outcome because this condition was actually included in the original panel for NBS in Bulgaria. The Bulgarian NBS program started in 1979, focusing on phenylketonuria and classical galactosemia. Nevertheless, the latter was discarded in 1989 due to “lack of effectiveness” [30,31]. To the best of our knowledge, there are no further publications or official statements on NBS for classical galactosemia in Bulgaria, so we could not elaborate more on that question.

### 4.2. Measures to Improve the Outcomes of Universal NBS Programs

We found raising awareness among prospective parents and society to be the most highly appraised measure to improve the outcomes of the current universal NBS program in Bulgaria. It came slightly ahead of increasing government funding in terms of the approval rate. This step has several dimensions. The most obvious one is to provide timely education so couples can better understand NBS and make informed choices [32,33]. Good-quality information in the prenatal period is recommended as an integral part of any NBS program [34,35]. Recently, experts have stressed the need to expand the methods for communication about NBS, accounting for the preferences of today’s generation of parents [36].

The other dimension of this measure is equally important. This is about engaging citizens and society in formulating and implementing NBS policy [37]. The issue of NBS expansion has historically frequently taken place after ad hoc consideration of conditions rather than in a planned and open manner [38]. At present, health policy operates in a different environment. Greater public involvement would lead to more representative policymaking and improved NBS health services [37]. In 2015, the Netherlands decided to expand its NBS panel from 17 to 31 conditions by implementing a rigorous framework and a transparent stakeholder process, setting an example for other countries as well [39].

Funding is a key issue for NBS programs, and particularly for their potential expansion. A clear majority of our study’s participants supported the idea of adding more conditions for universal NBS in Bulgaria. Such an enterprise would require substantial public investment. NBS is not only a laboratory test but a sequence of coordinated and harmonized health services from diagnosis to treatment and follow-up [40]. To qualify for public funding, NBS programs should meet certain criteria for cost-effectiveness, cost-benefit, and budget impact. Those requirements could be especially challenging for small countries like Bulgaria [7].

Over the last decade, interest in health economic evaluations of NBS programs has risen [41,42]. A decision to fund and implement a new NBS program requires complex organizational and infrastructure arrangements to be made [41]. Health economic assessments aim to better inform decision-makers and politicians about the balance of benefits and harms of NBS [7]. A systematic review of the Organization for Economic Co-operation and Development (OECD) countries identified several sources of potential costs and effects to be considered in the economic evaluation of NBS programs; they not only include the domains of diagnosis, treatment, and follow-up services, but also fields like overdiagnosis, pregnancy loss, and spillover effects on family members [42].

It is difficult to compare the findings and conclusions of the published economic evaluations of NBS programs from different jurisdictions because of the underlying variation in methodologies and health system settings [42]. Nevertheless, there are three recurring recommendations. First, NBS promotes the early diagnosis and onset of treatment, resulting in improved health outcomes and prognosis [28]. Second, the continuing advances in medical science and health technologies lead to better and cheaper NBS tools [43]. Third, NBS is also associated with important indirect savings due to reductions in productivity losses and consumption of public services [44].

Finally, our study explored the prospects of NBS expansion in Bulgaria in terms of specific conditions. We did not address the question of the technology that would be necessary for this potential endeavor. The introduction of novel genomic technologies into routine NBS screening is already taking place in some form [43,45,46]. Coupled with artificial intelligence, this creates a great window of opportunity for NBS [47]. Nevertheless, this anticipated development must be cautious and balanced in order to harness the potential of these new advances while maximizing the benefits and minimizing the risks of NBS activities [10]. Prior research must be conducted to determine and analyze the ethical, legal, organizational, and social repercussions [48].

### 4.3. Limitations

Our research has a number of limitations. First, we used purposive sampling for an online survey. Therefore, our findings may not be fully representative of all Bulgarian medical specialists. Nevertheless, we specifically targeted professionals who are affiliated with university hospitals and scientific societies. This approach generated a highly qualified sample of participants who are likely to inform and influence NBS policy in Bulgaria.

Second, we did not study the technological and financial challenges that would inevitably arise when expanding NBS in Bulgaria. These questions are equally important and would eventually shape the final political decision regarding which conditions should be added to the NBS panel in the country. More research and, in particular, country-specific economic evaluations are necessary to understand the complex nature of NBS policymaking.

Third, we only focused on the views and attitudes of specialists in pediatrics, neonatology, medical genetics, and biochemistry. In practice, NBS policymaking will be based on input from various stakeholders, including the government, other healthcare professionals, payers, ethicists, and patient organizations. It would be helpful to explore the attitudes and expectations of these specific groups and how they would interact in the process of NBS policymaking.

## 5. Conclusions

We found high support for adding more conditions to the panel for universal NBS in Bulgaria, with cystic fibrosis, thalassemia, and spinal muscular atrophy being the most obvious choices. In general, raising awareness among prospective parents and society and increasing government funding are perceived as the most important policy measures to enhance NBS activities in the country. Country-specific economic evaluations and research on the views of other stakeholders, including the government, payers, and patient organizations, are necessary to better understand and manage the complex nature of NBS policymaking.

## Figures and Tables

**Table 1:** Socio-demographic and career profile of the respondents.

Characteristic	*n* (%)
**Gender**	
Female	124 (80.5)
Male	30 (19.5)
**Mean age in years (±SD)**	49.7 ± 12.4
**Highest educational degree**	
M.Sc.	52 (33.8)
Ph.D.	83 (53.9)
D.Sc.	19 (12.3)
**Medical specialty (multiple responses allowed)**	
Medical genetics	22 (14.3)
Pediatrics	105 (68.2)
Pediatric gastroenterology	10 (6.5)
Pediatric endocrinology and metabolic diseases	23 (14.9)
Pediatric cardiology	4 (2.6)
Pediatric clinical hematology and oncology	8 (5.2)
Pediatric neurology	10 (6.5)
Pediatric nephrology and hemodialysis	4 (2.6)
Pediatric pneumology and phthisiology	15 (9.7)
Pediatric rheumatology	4 (2.6)
Neonatology	12 (7.8)
Biochemistry	4 (2.6)
Other	21 (13.6)
**Mean professional experience in years (±SD)**	21.5 ± 13.2
**Main professional role (>50% of the time)**	
Diagnosis and treatment	129 (83.8)
Teaching	15 (9.7)
Research	6 (3.4)
Administration	2 (1.3)
Other	2 (1.3)

**Table 2:** Knowledge and attitudes towards the current NBS program in Bulgaria (*n* = 154).

Topic	*n* (%)
**Self-rated knowledge about the current universal NBS program on a 1–5 scale (1 being the lowest and 5 being the highest)**	
1	2 (1.3)
2	5 (3.2)
3	30 (19.5)
4	70 (45.5)
5	47 (30.5)
**Participation in activities that are related to the current NBS program (multiple responses allowed)**	
Collection of samples from newborns	13 (8.4)
Primary processing and analysis of the collected samples	5 (3.2)
Confirmation of diagnosis	28 (18.2)
Treatment and follow-up of patients	48 (31.2)
Maintenance of epidemiological registers	2 (1.3)
Administration and control	7 (4.5)
None	85 (55.2)
**Assessment of the outcomes (coverage of all newborns and early diagnosis) of the current universal NBS program on a 1–5 scale (1 being the lowest and 5 being the highest)**	
1	1 (0.6)
2	9 (5.8)
3	30 (19.5)
4	75 (48.7)
5	39 (25.3)
**Expansion of the current NBS program by including additional disorders to screen**	
Yes	150 (97.4)
No	4 (2.6)

**Table 3:** Rationale of NBS inclusion for cystic fibrosis, thalassemia, and spinal muscular atrophy.

Wilson–Jungner Principle	Cystic Fibrosis (*n* = 134)	Thalassemia (*n* = 112)	Spinal Muscular Atrophy (*n* = 101)
The condition sought should be an important health problem	117 (87.3%)	94 (83.9%)	91 (90.1%)
There should be an accepted treatment for patients with recognized disease	82 (61.2%)	74 (66.1%)	50 (49.5%)
Facilities for diagnosis and treatment should be available	58 (43.3%)	61 (54.5%)	43 (42.6%)
There should be a recognizable latent or early symptomatic stage	67 (50.0%)	57 (50.9%)	60 (59.4%)
There should be a suitable test or examination	92 (68.7%)	71 (63.4%)	45 (44.6%)
The test should be acceptable to the population	55 (41.0%)	51 (45.5%)	24 (23.8%)
The natural history of the condition, including development from latent to declared disease, should be adequately understood	46 (34.3%)	51 (45.5%)	44 (43.6%)
There should be an agreed policy on whom to treat as patients	64 (47.8%)	62 (55.4%)	31 (30.7%)
The cost of case-finding (including diagnosis and treatment of patients diagnosed) should be economically balanced in relation to possible expenditure on medical care as a whole	40 (29.9%)	37 (33.0%)	17 (16.8%)
Case-finding should be a continuing process and not a “once and for all” project	71 (53.0%)	51 (45.5%)	47 (46.5%)

## Data Availability

The datasets used and analyzed during the current study are available from the corresponding author on reasonable request.

## Supplementary Materials

The following supporting information can be downloaded at: https://www.mdpi.com/article/10.3390/ijns9040057/s1, Supplementary File S1: List of 35 prospective disorders to be included in the panel for universal NBS in Bulgaria (based on [9]), Supplementary File S2: Approval of inclusion in the panel for universal NBS in Bulgaria for the disorders studied (the full list is based on [9]), Supplementary File S3: Checklist for Reporting Results of Internet E-Surveys (CHERRIES).
