# Supplementary material for: Prospects for Expansion of Universal Newborn Screening in Bulgaria: A Survey among Medical Professionals
Source: Int J Neonatal Screen. 2023 Oct 11;9(4):57. doi: 10.3390/ijns9040057 (PMC10594438; doi:10.3390/ijns9040057)
Supplement: Supplementary file 1 [file IJNS-09-00057-s001.zip › Supplementary File S1.pdf]

Supplementary file S1. List of 35 prospective disorders to be included in the panel for universal NBS in Bulgaria (based on Loeber JG, Platis D, Zetterström RH, Almashanu S, Boemer F, Bonham JR, et al. Neonatal Screening in Europe Revisited: An ISNS Perspective on the Current State and Developments Since 2010. Int J Neonatal Screen. 2021 Mar 5;7(1):15.)

1. 3-Hydroxy-3-methylglutaric aciduria
2. 3-Methylcrotonyl-CoA carboxylase deficiency/3-Methylglutacon aciduria/2-methyl-3-OH-butyric aciduria
3. Argininemia
4. Argininosuccinic aciduria
5. Beta-ketothiolase deficiency
6. Biotinidase deficiency
7. Carnitine acylcarnitine translocase deficiency
8. Carnitine palmitoyltransferase deficiency type I
9. Carnitine palmitoyltransferase type II / Carnitine acylcarnitine transporter deficiency
10. Carnitine uptake defect
11. Citrullinemia type I and II
12. Classical galactosemia
13. Cystic fibrosis
14. Glucose-6-phophate dehydrogenase deficiency
15. Glutaric acidemia type I
16. Glutaric acidemia type II / multiple acyl coA dehydrogenase deficiency
17. Holocarboxylase synthetase deficiency
18. Homocystinuria

19. Isovaleric acidemia

20. Long-chain L-3-hydroxyacyl-CoA dehydrogenase deficiency / Trifunctional protein deficiency

21. Maple syrup urine disease

22. Medium-chain acyl-CoA dehydrogenase deficiency

23. Methionine adenosyl transferase I/III deficiency

24. Methylmalonic acidemia

25. Multiple carboxylase deficiency

26. Propionic acidemia

27. Remethylation disorders (methylenetetrahydrofolate reductase, methylcobalamine deficiencies)

28. Severe combined immunodeficiencies

29. Short-chain acyl-CoA dehydrogenase deficiency

30. Spinal muscular atrophy

31. Thalassemia

32. Tyrosinemia type I

33. Tyrosinemia type II

34. Very long-chain acyl-CoA dehydrogenase deficiency

35. x-Adrenoleukodystrophy
