# Supplementary material for: Prospects for Expansion of Universal Newborn Screening in Bulgaria: A Survey among Medical Professionals
Source: Int J Neonatal Screen. 2023 Oct 11;9(4):57. doi: 10.3390/ijns9040057 (PMC10594438; doi:10.3390/ijns9040057)
Supplement: Supplementary file 1 [file IJNS-09-00057-s001.zip › Supplementary File S2.pdf]

Supplementary File S2. Approval of inclusion in the panel for universal NBS in Bulgaria for the disorders studied (the full list is based on Loeber JG, Platis D, Zetterström RH, Almashanu S, Boemer F, Bonham JR, et al. Neonatal Screening in Europe Revisited: An ISNS Perspective on the Current State and Developments Since 2010. Int J Neonatal Screen. 2021 Mar 5;7(1):15.)

Disorder / Group of disorders	Approval of inclusion in the panel for universal NBS in Bulgaria	Rate of approval (n = 154)
Cystic fibrosis	134	87.0%
Thalassemia	112	72.7%
Spinal muscular atrophy	101	65.6%
Classical galactosemia	91	59.1%
Severe combined immunodeficiencies	76	49.4%
Glucose-6-phophate dehydrogenase deficiency	72	46.8%
Maple syrup urine disease	59	38.3%
Homocystinuria	55	35.7%
Tyrosinemia type I	54	35.1%
Methylmalonic acidemia	48	31.2%
x-Adrenoleukodystrophy	42	27.3%
Tyrosinemia type II	41	26.6%
Propionic acidemia	40	26.0%
Biotinidase deficiency	39	25.3%
Isovaleric acidemia (IVA)/2-Methylbutyrylglycinuria	36	23.4%
Glutaric acidemia type I	35	22.7%
3-Hydroxy-3-methylglutaric aciduria	33	21.4%
Multiple carboxylase deficiency	33	21.4%
Citrullinemia type I / II	30	19.5%
Medium-chain acyl-CoA dehydrogenase deficiency	30	19.5%
Carnitine acylcarnitine translocase deficiency	29	18.8%
Carnitine uptake defect	28	18.2%
Argininemia	27	17.5%
Glutaric acidemia type II / multiple acyl coA dehydrogenase deficiency	27	17.5%

3-Methylcrotonyl-CoA carboxylase deficiency/3-Methylglutacon aciduria/2-methyl-3-OH-butyric aciduria	26	16.9%
Carnitine palmitoyltransferase type II/Carnitine acylcarnitine transporter deficiency	26	16.9%
Long-chain L-3-hydroxyacyl-CoA dehydrogenase deficiency/Trifunctional protein deficiency	26	16.9%
Very long-chain acyl-CoA dehydrogenase deficiency	26	16.9%
Beta-ketothiolase deficiency	25	16.2%
Carnitine palmitoyltransferase deficiency type I	25	16.2%
Argininosuccinic aciduria	23	14.9%
Short-chain acyl-CoA dehydrogenase deficiency	23	14.9%
Remethylation disorders (methylenetetrahydrofolate reductase, methylcobalamine deficiencies)	22	14.3%
Holocarboxylase synthetase deficiency	19	12.3%
Methionine adenosyl transferase I/III deficiency	18	11.7%
